# Cytotoxicity of naringenin induces Bax‐mediated mitochondrial apoptosis in human lung adenocarcinoma A549 cells

**DOI:** 10.1002/tox.23003

**Published:** 2020-07-15

**Authors:** Win‐Long Lu, Chang‐Tze Ricky Yu, Hsiu‐Man Lien, Gwo‐Tarng Sheu, Shur‐Hueih Cherng

**Affiliations:** ^1^ Institute of Medicine, Chung Shan Medical University Taichung Taiwan; ^2^ Department of Applied Chemistry National Chi Nan University Nantou Taiwan; ^3^ Department of Biotechnology Hung Kuang University Taichung city Taiwan; ^4^ Department of Medical Oncology and Chest Medicine Chung Shan Medical University Hospital Taichung Taiwan

**Keywords:** apoptosis, Bax, caspase, lung cancer, naringenin

## Abstract

Naringenin (NGEN), a natural flavonoid has growth inhibition and apoptosis‐inducing activities in several cancer cells. However, the cytotoxicity mechanisms of NGEN in cell death of lung cancer cells have not been fully defined. In present study, treatment of human lung adenocarcinoma A549 cells with NGEN resulted in time‐ and dose‐dependent decreases in cell viability. Moreover, NGEN significantly induced apoptosis evidenced by morphological changes, DAPI staining, TUNEL assay and sub‐G1 population increase. In NGEN‐treated cells, intensely upregulated Bax and down‐regulated Bcl‐2 proteins were detected and the Bax protein associated with the mitochondrial membrane was analyzed by subcellular fractionation. Knockdown of the Bax expression by the shRNA method dramatically protected A549 cells against NGEN‐induced apoptosis. Treatment with the inhibitors of caspase‐3, ‐8, or ‐9 significantly reduced NGEN‐induced apoptotic deaths. Taken together, our results demonstrate that NGEN‐induced apoptosis may occur via a Bax‐activated mitochondrial pathway in lung adenocarcinoma A549 cells.

## 1 INTRODUCTION

Naringenin (NGEN) is a flavanone, a type of flavonoid that is produced in citrus fruits in large quantities. NGEN has various beneficial activities including anti‐diabetic,^1^ antioxidant,^2^, ^3^ and anti‐inflammatory,^4^ and it has also provided neuroprotective activities in different experimental rodent models.^5^, ^6^ The in vitro, in vivo, and clinical trials reports have been nicely reviewed for NGEN therapeutic potential in medicine.^7^ NGEN increased cell viability in isolated neurons that were obtained from the brains of Sprague‐Dawley rats and decreased the rate of cell apoptosis.^8^ These findings demonstrate that NGEN eliminates oxidative stress and recovers from mitochondrial abnormality through the Nrf2/ARE signaling pathway activation in neurons.^8^ In contrast to the protection activities found in neuron, NGEN has also exhibited great chemopreventive potential against colon cancer in Wistar rats.^9^ Administration of NGEN to rats with induced gastric carcinoma has markedly enhanced redox status and decreased the risk of cancer.^10^ Therefore, in additional to the normal cell protection effect, NGEN also provides anti‐proliferation and apoptosis‐inducting activities for cancer cells. Moreover, NGEN induced apoptosis and enhanced reactive oxygen species (ROS) production in prostate cancer PC3 and LNCaP cells; in which, NGEN also induced mitochondrial membrane potential depletion, resulted in decreased Bcl‐2 and increased Bax proteins in PC3 cells only, but similar results had not been found in LNCaP cells.^11^

Mitochondria‐mediated apoptosis is mediated by the Bcl‐2 proteins family, which can strengthen (eg, Bax and Bid) or impede (eg, Bcl‐2 and Bcl‐xL) apoptosis.^12^, ^13^ Bcl‐2 was originally found in a translocation chromosomal fragment in B‐cell lymphoma and has been proven to be a proto‐oncogene.^14^, ^15^ The members of the Bcl‐2 family are separated into three major groups: the first is made of antiapoptotic proteins, including Bcl‐2, Bcl‐w, Bcl‐xL, and Mcl‐1. The second group has been classified as proapoptotic proteins, such as Bax, Bak and PUMA. The third group is named the apoptosis initiator proteins including Bad, Bid, Bim, PUMA, and NOXA and is made of BH3 domain‐only.^16^ In case of no apoptotic stress, Bcl‐2 and Bcl‐xL interact into heterodimers with Bax and Bak (proapoptotic) to preserve the integrity of the outer mitochondrial membrane and restraint mitochondrial apoptosis. However, when apoptotic stimulation occurs, it changes the ratios between antiapoptotic and proapoptotic groups, driving to the construction of Bax/Bak complexes followed by punching through the membrane of mitochondria. In previous research, it has been demonstrated that the cytochrome c was discharged from the mitochondria to the cytosol through the opened orifices and it formed an apoptosis multi‐units with procaspase‐9, apoptotic protease activation factor‐1 (Apaf‐1), and deoxyadenosine triphosphate (dATP) to activate caspase‐9 and caspase‐3 mediated apoptosis.^16^, ^17^

The cytotoxic effects of NGEN have been identified in different human cancer cell lines, including human lung cancer cells. Treatment of human lung cancer A549 cells with modified NGEN derivatives, including 7‐O‐benzyl NGEN (KUF‐1) and KUF‐7 induced significant apoptosis and intracellular ROS production.^18^ In mice with pulmonary fibrosis, NGEN also significantly reduced lung metastases and increased their survival by repressing transforming growth factor‐β1 and reducing regulatory T cells that might improve the immunosuppressive environment. Therefore, it has been suggested that NGEN could be used as a therapeutic agent to control lung cancer and lung fibrosis.^19^ Treatment with a low dosage of NGEN combined with TRAIL induced apoptosis in TRAIL‐resistant lung cancer cells by upregulating death receptor 5 also indicated NGEN might sensitizing extrinsic death pathway.^20^ Furthermore, NGEN also inhibited the AKT signaling pathway and reduced MMP‐2 and ‐9 activities to inhibit the migration of A549 cells.^21^

Both anti‐proliferation and apoptosis induction are significant functions of the response of cancers to chemotherapeutic agents. Therefore, it is crucially important to characterize the molecular mechanism of NGEN‐induced effects. In present study, the human lung carcinoma cell line A549 was used to evaluate the molecular mechanism of NGEN‐induced apoptosis. Our results may help to understand whether NGEN possesses therapeutic potential for lung cancer management.

## 2 MATERIALS AND METHODS

### 2.1 Chemicals

NGEN (2,3‐dihydro‐5,7‐dihydroxy‐2‐[4‐hydroxyphenyl]‐4H‐1‐benzopyran‐4‐one), dithiothreitol (DTT), 4′,6‐diamindino‐2‐phenylinodole (DAPI), EGTA, leupeptin, and Triton X‐100 were bought from Sigma‐Aldrich Co. (St Louis, MO). Anti‐Bcl‐2 (sc‐7328), anti‐Bcl‐xL (sc‐7195), anti‐Bad (sc‐7869), and anti‐Bax (sc‐493) were purchased from Santa Cruz Biotechnology (Santa Cruz, CA). Anti‐cytochrome c (Cat. No. 556432) was bought from PharMingen (San Diego, CA). Anti‐β‐Actin (A5441) was purchased from Sigma‐Aldrich Co. and anti‐PUMA (Cat. No. PC686) was bought from Oncogene Science, Inc. (Uniondale, NY). Anti‐cytochrome oxidase IV (Cat. No. 556423) was purchased from Molecular Probes (Eugene, OR). Secondary antibodies of peroxidase‐conjugated anti‐rabbit IgG (Cat. No. NA934), anti‐mouse (Cat. No. NA931) and the enhanced chemiluminescence (ECL) detection kits were obtained from Amersham Life Science (Buckinghamshire, UK). Inhibitors of Caspase‐3 (z‐DEVD‐fmk), caspase‐8 (z‐IETD‐fmk), and caspase‐9 (z‐LEHD‐fmk) were obtained from KAMIYA Biomedical Company (Seattle, WA). Reagents of caspase activity assay were purchased from R&D Systems (Minneapolis, MN). All other chemicals with analytical grade quality had been obtained through commercial sources.

### 2.2 Cell culture and Trypan blue viability test

The human lung A549 cancer cell line that carries a wild‐type p53 gene was bought from the American Type Culture Collection (Rockville, MD) and the cell line has been further authenticated by Mission Biotech (Taipei, Taiwan) with cell line STR locus DNA typing (Case number CID20150026) that matches all repeat numbers of the A549 profile (ATCC CCL‐185). Cells were cultured in RPMI‐1640 medium (Gibco‐BRL, Gaithersburg, MD) added with 10% heat‐inactivated fetal bovine serum (Hyclone, Logan, Utah), antibiotics (100 U/mL penicillin and 100 μg/mL streptomycin), and 2 mM glutamine, at 37°C in a humidified incubator containing 95% air and 5% CO_2_. For growth inhibition assay, cells were cultivated into 12‐well plates and incubated (24 hours), and then cells were treated with indicated concentrations of NGEN. The quantity of viable cells was counted by the Trypan blue dye exclusion method as previously described.^22^ Briefly, cells were collected, and then incubation with 0.4% Trypan blue solution. The viable cells were then counted under a microscope using a hematocytometer. The viable cells (average percentage) were calculated from three independent experiments.

### 2.3 Inhibition of Bax by VSV‐G pseudo lentivirus‐shRNA

Initially, lentivirus vectors at a multiplicity of infection of 2 were used to infect A549 cells followed by 24 hours incubation. To harvest stably infected cells, the cells were selected by puromycin (2 μg/mL, Sigma, P8833) with a fresh medium and incubated for 6 days continuously until all parental A549 cells died. The shLUC and shBax cells were used for further analysis. Lung cancer cells (8 × 10^4^/well) were seeded onto 24‐well plates and incubated at 37°C for 24 hours, then cells were treated with NGEN (800 μM) for 24 hours. The number of viable cells was determined by the Trypan blue dye exclusion method. We purchased RNAi reagents from the National RNAi Core Facility (Institute of Molecular Biology/Genomic Research Center, Academia Sinica, Taiwan). The unique TRC number of each clone as following: shLuc TRCN0000072249 targeted to luciferase for vector control; clones of shBax (TRCN0000312625) targeted to Bax.

### 2.4 Propidium iodide (PI) staining for sub‐G1 analysis

Apoptotic cells were measured through flow cytometry by the detection of fragmentized DNA. Briefly, 1 × 10^6^ NGEN‐treated cells were harvested, followed by washing with phosphate‐buffered saline (PBS), and then fixed in 80% ethanol. The cells were washed with PBS once and then incubated at 37°C for 30 minutes with a reagent mixture containing 100 μg/mL RNase plus 50 μg/mL PI. Stained cells were examined on a FACScan flow cytometer (Becton Dickinson, San Jose, CA). The apoptotic cells (% of sub‐G1 population) were calculated using ModFITLT 2.0 software.

### 2.5 Detection of DNA fragmentation in apoptotic cells by DAPI staining and TUNEL assay

NGEN (800 μM) were added to A549 cells for 48 hours, followed by washing with PBS. To quantify apoptotic cells, harvested cells were settled in 2% paraformaldehyde for 30 minutes followed by permeabilizing at room temperature with 0.1% Triton X‐100/PBS (30 minutes). Later, PBS‐washed cells were treated with terminal deoxynucleotidyl transferase dUTP nick end labeling (TUNEL) assay reagents (Boehringer Mannheim, Germany). In brief, cells were exposed to TUNEL reaction buffer (supplied by the manufacturer) in the dark at 37°C for 1 hour for labeling. Those cells were washed with PBS subsequently, and the results were obtained by a fluorescence microscope. Fluorescein‐positive cells were counted as DNA‐fragmented, apoptotic cells. On the other hand, fixed cells were treated with DAPI (1 mg/mL) at 37°C for 10 minutes that also observed by a fluorescence microscope.

### 2.6 Caspase activity assay

Cell lysates of NGEN‐treated or untreated A549 cells were collected and evaluated for caspase‐3 (DEVD‐AFC), caspase‐8 (IETD‐AFC), and caspase‐9 (LEHD‐AFC) activities with a caspase‐specific peptide substrate respectively, that conjugated with the molecule (7‐amino‐4‐trifluoromethl coumarin) as fluorescent reporter (R&D systems, Minneapolis, MN). When peptide is cleaved by the caspase, the released fluorochrome would be stimulated by light (400 nm), generating fluorescence at 505 nm. Therefore, the amount of caspase enzymatic activity in the cell lysate was positively correlated with the fluorescence signal level that was obtained with a fluorescent microplate reader (Fluoro shan Ascent, Labsystems, Finland).

### 2.7 Protein preparation and immunoblotting

Cells were treated with or without NGEN at various times. After treatment, both floating and adherent cells were collected, washed with PBS, and lysed in modified RIPA buffer (50 mM Tris, pH 7.4, 1 mM EGTA, 5 mM EDTA, 150 mM NaCl, 5 mg/mL leupeptin, 0.2 mM phenylmethylsulfonylfluorid [PMSF], 5 μg/mL aprotinin, 1% Triton X‐100, 0.25% sodium deoxycholate, 1 mM sodium fluoride, 1 mM sodium orthovanadate, and 1 mM DTT). Protein concentration was measured by the Bradford method. To perform Western blot analysis, comparable amounts of total protein were loaded onto SDS‐polyacrylamide gels and separated by electrophoresis. Later, the proteins on gel were transferred onto a PVDF membrane electrophoretically (PerkinElmer Life Sciences, MA). Immunoblots were further characterized with individual primary antibodies and then processed by a secondary antibody; proteins‐antibodies reactions were visualized by using an ECL kit (Amersham Life Science) according to the manufacturer's instructions.

### 2.8 Preparation of subcellular fractions

The procedures of cell fractionation have been described previously.^23^ Concisely, cells were harvested at indicated time and followed by washing with PBS then stored at −80°C. The pellets were then thawed at 4°C and suspended in cytosol extraction buffer (20 mM HEPES, pH 7.5, 5 μg/mL leupeptin, 5 μg/mL aprotinin, 1 mM PMSF, 1 mM DTT 1 mM EDTA, 1 mM EGTA, 1.5 mM MgCl_2_, 10 mM KCl) at 4°C. Cell lysates was centrifuged at 100 000*g* (30 minutes) at 4°C to obtain the supernatant as the cytosolic fraction. The remained pellet was further suspended extensively in modified RIPA buffer at 4°C followed by centrifugation to collect the supernatant as the particulate fraction. The particulate fraction includes membrane‐organelle associated proteins that represent mitochondria content. Protein concentrations were further determined by Bradford method and samples were applied to subsequent experiments.

### 2.9 Statistical analysis

The calculated data from three separate experiments are indicated as mean ± SD Statistical differences were examined by the Student's *t* test. Multiple groups (viability test, caspase activity and inhibition) were compared using one‐way ANOVA with Dunnett post‐hoc test using SPSS version 12 (Chicago, IL) and considered significant at the *P < .05, **P < .01, or ***P < .001 level.

## 3 RESULTS

### 3.1 NGEN reduces the viability of A549 cells in time and dose‐dependent manner

**FIGURE 1:**
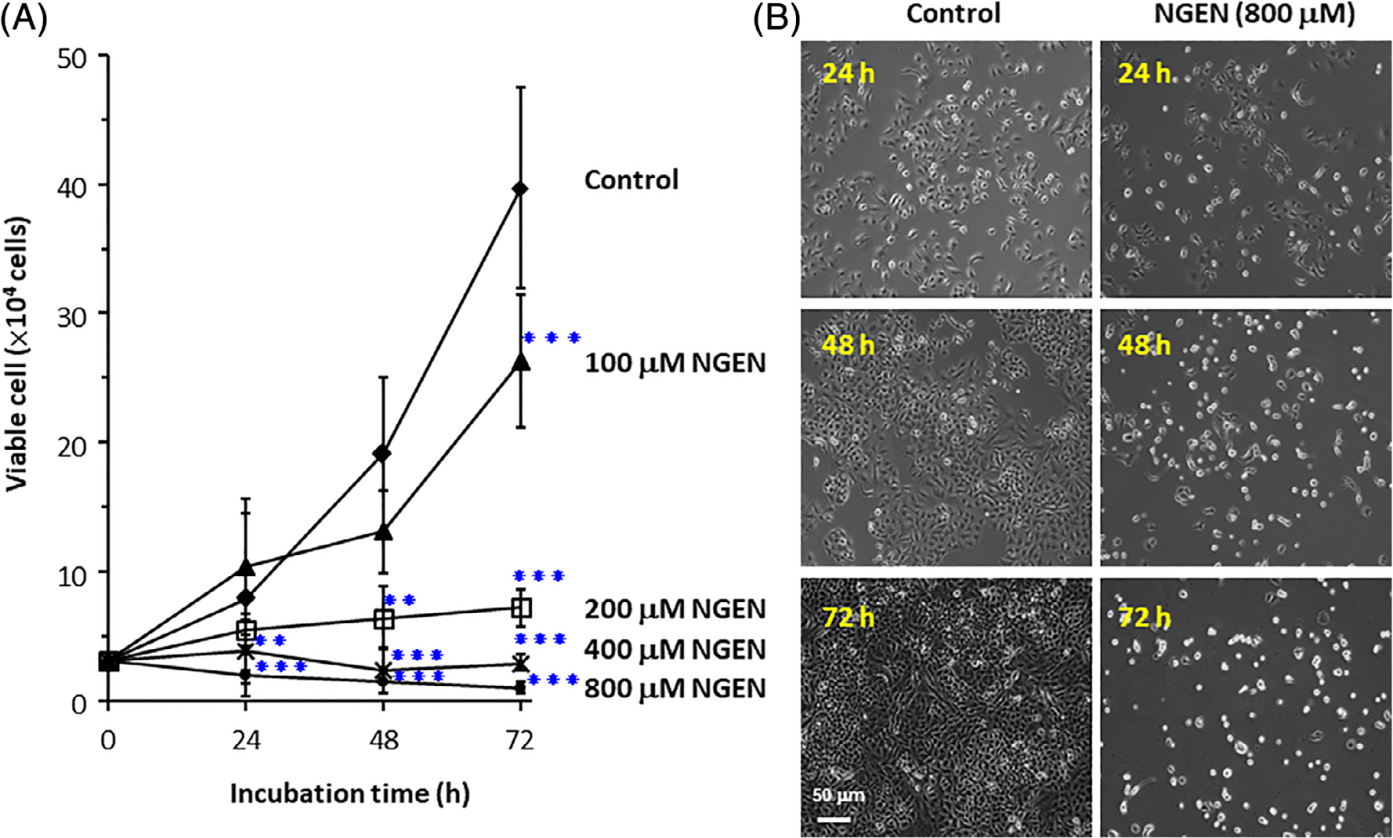
Determination of NGEN sensitivity by Trypan blue viability assay. A, Cells were seeded into 12‐well plates. After 24 hours, cells were treated with various concentrations of NGEN (100, 200 400, and 800 μM) for the indicated time. The number of viable cells was determined by the Trypan blue dye exclusion method. Comparisons were performed using one‐way ANOVA with Dunnett post‐hoc test and considered significant at the **P < .01, or ***P < .001 level. B, Cell morphology was obtained by an Olympus IX 70 microscope with magnification of ×100 (scale bar, 50 μm). All the treatments were done in triplicate [Color figure can be viewed at wileyonlinelibrary.com]

To analyze the cytotoxicity of NGEN in A549 cells, cells were treated with various concentrations (100‐800 μM) of NGEN for 24, 48, and 72 hours. When 100 and 200 μM of NGEN were used, the growth inhibition effect was observed with 24 hours exposure. When A549 cells were exposed with 400 and 800 μM of NGEN for 48 hours, viabilities of the cells were significantly reduced, indicating that cell death may be involved (Figure 1A). The morphologies of A549 cells are compared in Figure 1B, which shows that more dense and round cells were observed in A549 cells treated with NGEN (800 μM) after 48 and 72 hours of exposure.

### 3.2 NGEN induces DNA fragmentation and apoptosis of A549 cells

**FIGURE 2:**
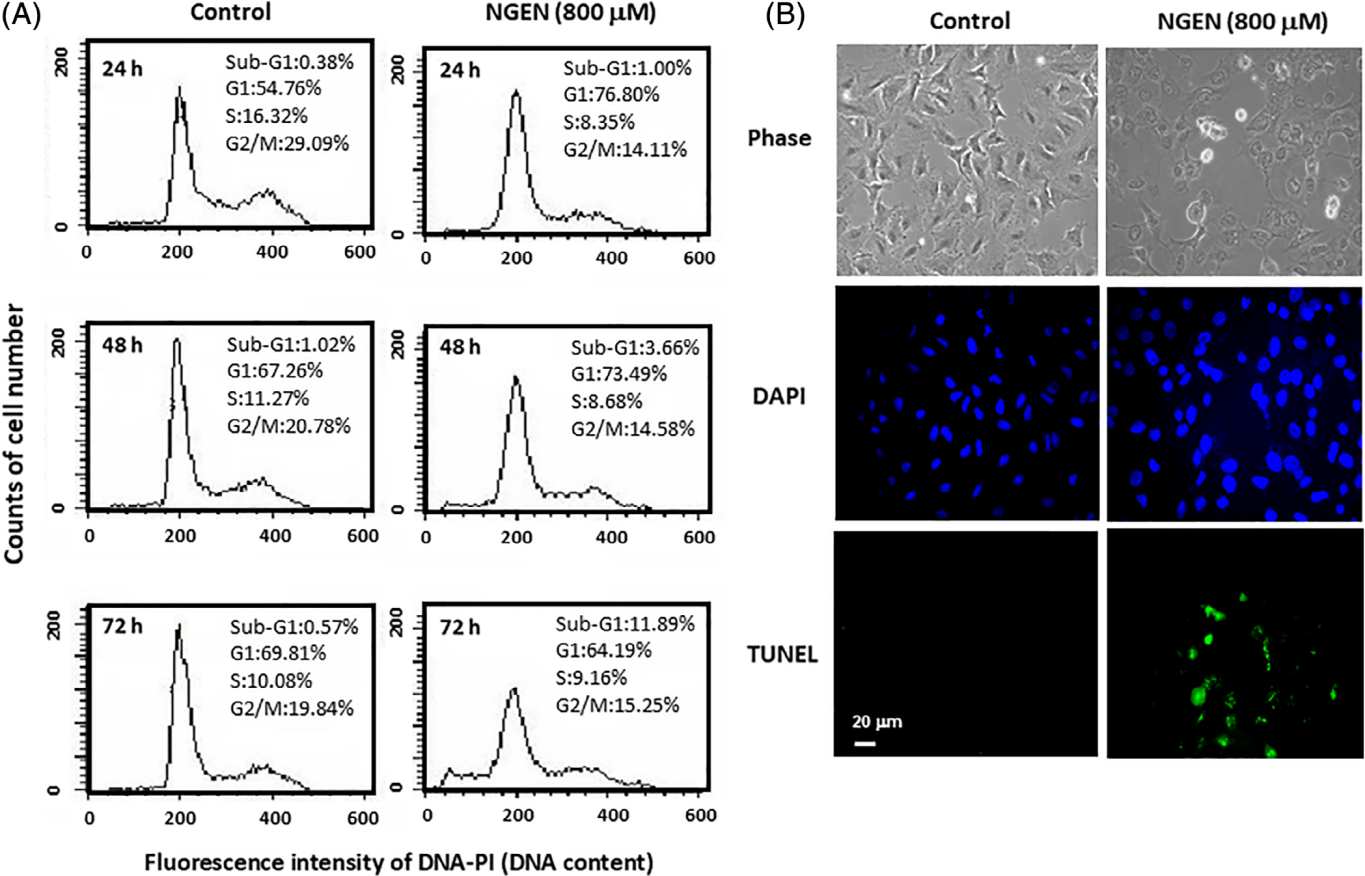
NGEN induces DNA fragmentation as detected by flow cytometry and TUNEL assay. A, The cells were treated with 800 μM NGEN for 24, 48, and 72 hours, and were harvested for flow cytometry analysis of the sub‐G1 population. Quantification of sub‐G1 phase and cell population in each phase was determined using ModFITLT 2.0 software. B, A549 cells were treated without or with 800 μM of NGEN for 48 hours, and then TUNEL assay was performed, and nuclear DNA was stained using DAPI. The stained cells were examined by a fluorescence microscope with a magnification of ×200 (scale bar, 20 μm) [Color figure can be viewed at wileyonlinelibrary.com]

When cells suffering from apoptosis, they lose part of their DNA as a result of DNA fragmentation in later apoptosis. One of the assays to determine apoptosis by flow cytometry is the estimation of fractional DNA content to quantitate apoptosis. Using flow cytometry, part of propidium iodide (PI) stained cells stain less intensely and show a peak below the G1 peak, which is identified as the sub‐G1 peak.^24^ When A549 cells were treated with NGEN, the sub‐G1 peak was increased from 3.66% to 11.89% after 48 and 72 hours of exposure, respectively (Figure 2A). Furthermore, the margin of the nucleus is abnormal and the condensed chromosome is easily stained in the NGEN‐treated apoptotic cells detected by DAPI staining. The DNA fragmentation of NGEN‐treated cells was also observed by TUNEL assay (Figure 2B), showing that NGEN induces A549 cell death via an apoptosis associated death pathway.

### 3.3 NGEN induces caspase‐dependent apoptotic cell death

**FIGURE 3:**
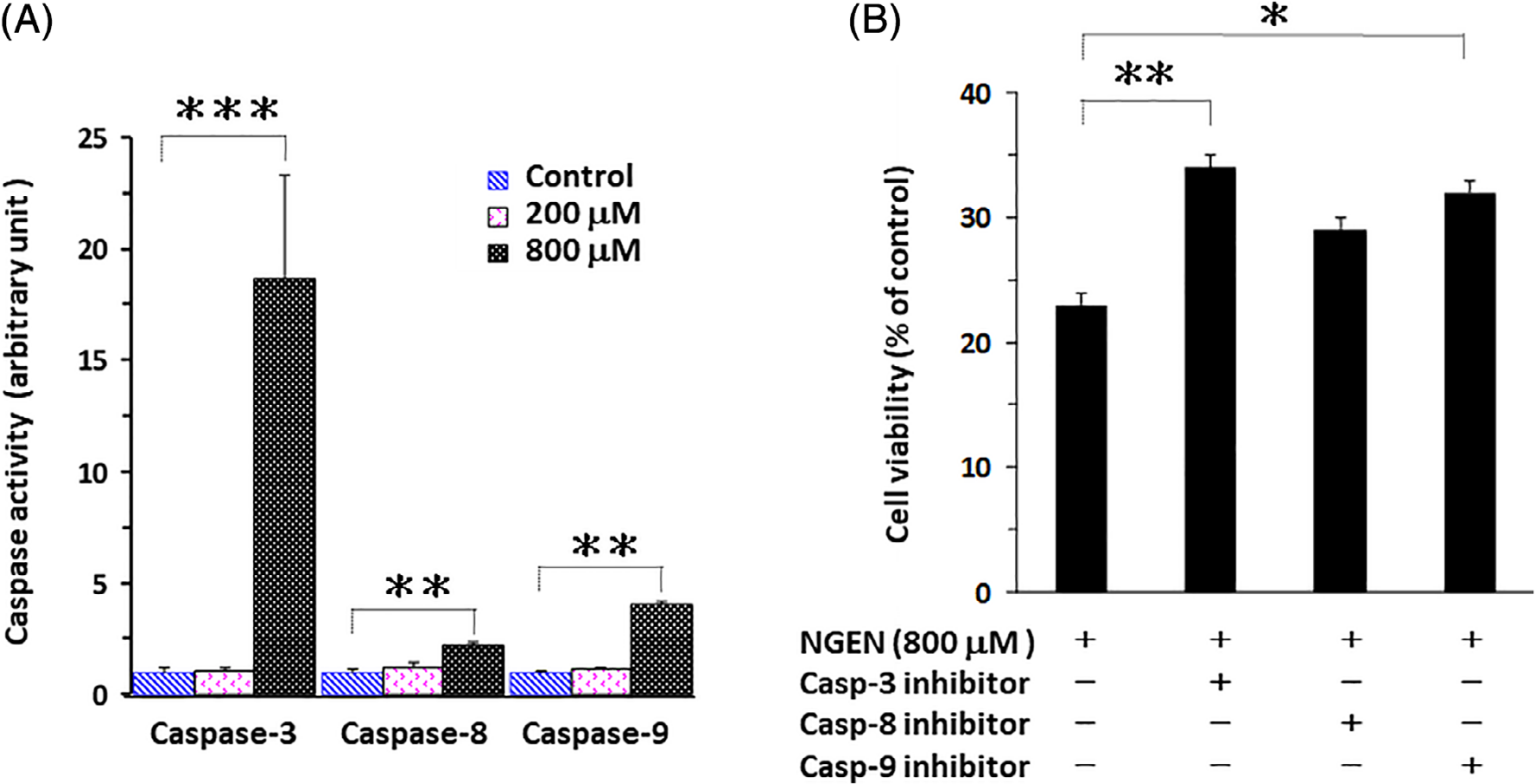
NGEN activates caspases‐mediated cell death. A, A549 cells were treated with or without NGEN (200 or 800 μM) for 24 hours. Extracts from untreated or NGEN‐treated cells were assayed for caspase activities using fluorogenic peptide substrates. B, A549 cells were treated with NGEN (800 μM) after pretreatment of the caspase‐3 (z‐DEVD‐fmk), caspase‐8 (z‐IETD‐fmk), and caspase‐9 (z‐LEHD‐fmk) inhibitors (100 μM) respectively for 1 hour. After 24 hours treatment, cell numbers were calculated using hemocytometers by the Trypan blue dye exclusion method. Comparisons were performed using one‐way ANOVA with Dunnett post hoc test and considered significant at the *P < .05, **P < .01, or ***P < .001 level [Color figure can be viewed at wileyonlinelibrary.com]

The mechanism of NGEN‐induced apoptosis was analyzed by caspase activity assay to distinguish the association of unique caspases in NGEN‐induced cell death. Significant caspase‐3 activity was detected and caspase‐9 was also activated in cells treated with 800 μM of NGEN (Figure 3A). To assess the contribution of caspases in NGEN‐induced cell death, the caspase‐3 inhibitor, caspase‐8 inhibitor, and caspase‐9 inhibitor were applied individually in combination with NGEN, followed by counting of viable cells. The data showed caspase‐3, caspase‐9 and caspase‐8 inhibitors mitigate NGEN‐induced cell death with respective efficacy (Figure 3B). According to these results, the intrinsic apoptosis through caspase‐9 activated capspase‐3 plays the major role in NGEN‐induced apoptotic death; however, the extrinsic apoptosis via caspase‐8 activation is not affected as much as the intrinsic pathway.

### 3.4 NGEN upregulates Bax and PUMA proapoptotic protein expression

**FIGURE 4:**
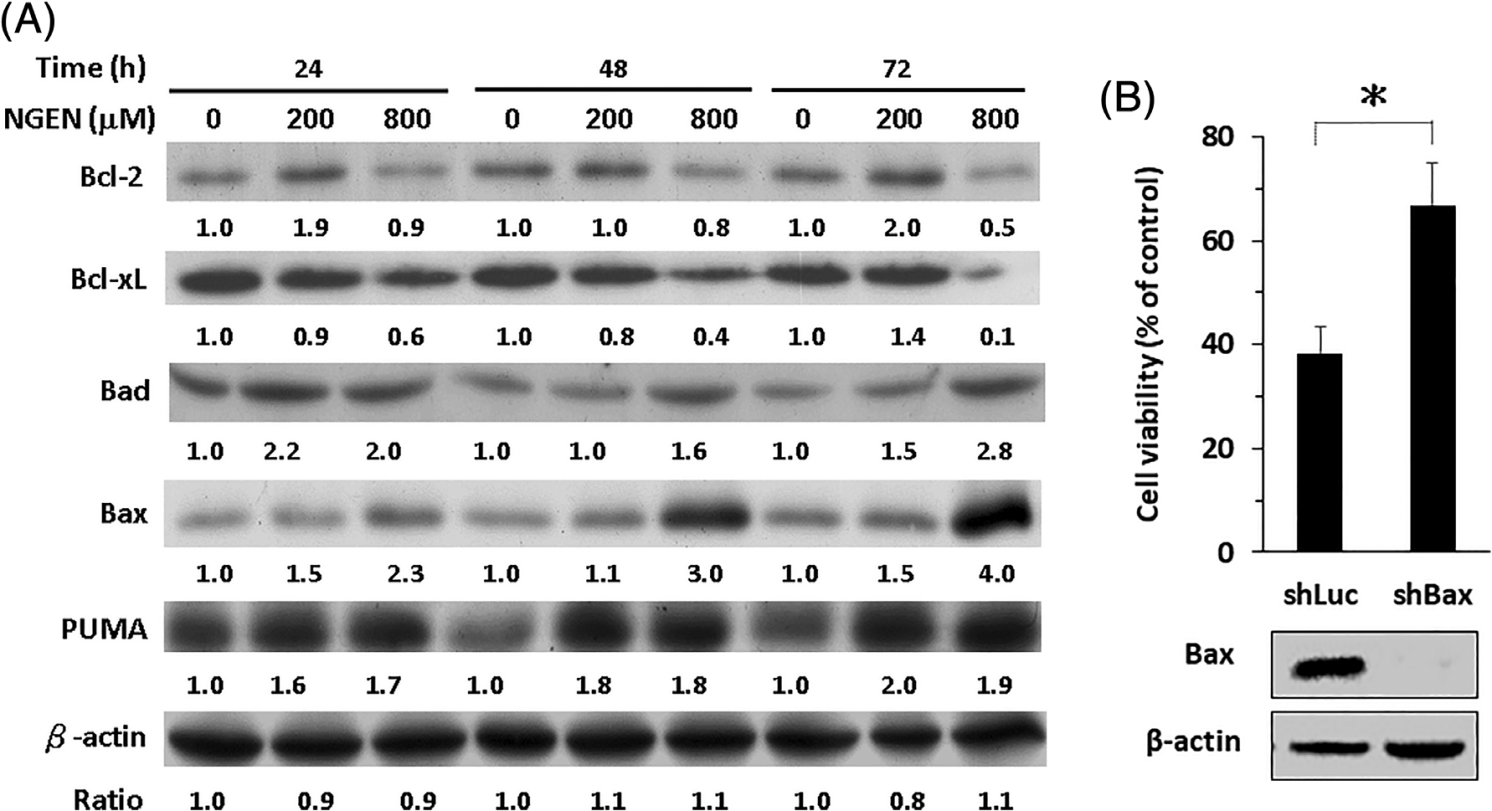
Characterization of NGEN regulated proteins associated with apoptosis. A, A549 cells were treated without or with NGEN (200 or 800 μM) for 24, 48, and 72 hours. After treatment, cells lyses were extracted, and the levels of Bcl‐2 family proteins were analyzed by Western blot. B, A549‐shLuc cells and A549‐shBax cells were treated with 800 μM of NGEN for 24 hours. Cell viability was measured using direct cell counting by the Trypan blue dye exclusion method (upper panel). The expression of Bax was analyzed by Western blot (lower panel). Comparison was performed using Student's *t* test and considered significant at the *P < .05. Protein intensities were calculated using ImageJ (https://imagej.nih.gov/ij/) and the folds of change were as listed

The induction of apoptosis by NGEN through an intrinsic pathway was further investigated by Western blot assay. The expression levels of antiapoptotic Bcl‐2, Bcl‐xL, proapoptotic Bax, PUMA and the apoptosis initiator Bad were examined with treatment of NGEN after 24, 48, and 72 hours (Figure 4A). The data showed that NGEN downregulates Bcl‐2 and Bcl‐xL expressions. Meanwhile, enhanced Bad, Bax and PUMA expressions were detected under 800 μM NGEN treatment of A549 cells. The significant interactions of the Bcl‐2 family could be observed when cells were treated with 800 μM NGEN for 72 hours. PUMA is a p53‐regulated protein that introduces death messages mostly to the mitochondria, where it works indirectly on the Bcl‐2 family members Bax and/or Bak by alleviating the inhibition that have been assessed by antiapoptotic members.^25^ PUMA expression was enhanced before Bax upregulation (Figure 4A, 48 and 72 hours), supporting this early conclusion. Interestingly, a cleaved form of Bad was also detected when cells were treated with 800 μM NGEN. Although Bad may not directly activate apoptosis, it reduces the threshold at which apoptosis is introduced.^26^ Human Bad is processed by a caspase when exposure of Jurkat T cells to the anti‐FAS antibody. Moreover, a cleaved form of human Bad lacking N‐terminal 28 amino acids was suggested to induce apoptosis more significantly than wild‐type Bad.^27^ When the intrinsic apoptotic pathway is overwhelming, the procaspase‐3 is activated by caspase‐9 resulting in apoptosis. The contribution of Bax upregulation in NGEN‐induced apoptosis was further evaluated by a sh‐Bax knockdown method (Figure 4B). We compared the viability of A549‐shLUC cells with the A549‐shBax cells by NGEN (800 μM) treatment for 24 hours. The survival of NGEN‐treated shBax cells was significantly increased (Figure 4B upper panel) when Bax expression was inhibited (Figure 4B lower panel). Our data demonstrated that high doses of NGEN induce mitochondria‐mediated apoptosis via Bax upregulation.

### 3.5 Mitochondrial translocation of Bax and cytochrome c release were induced by NGEN

**FIGURE 5:**
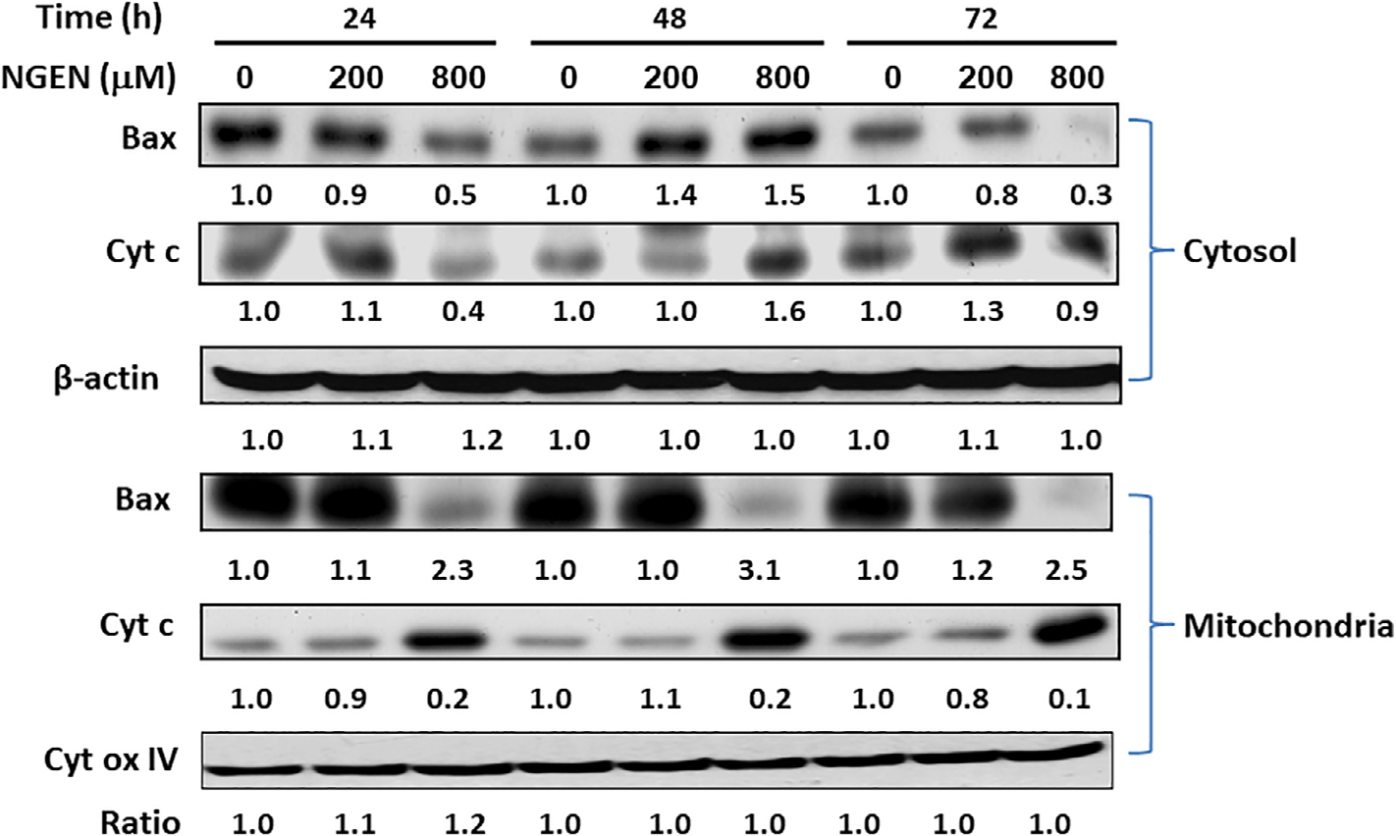
Analysis of Bax and cytochrome c levels in cytosolic and membrane fractions. A549 cells were treated without or with NGEN (200 or 800 μM) for 24, 48, and 72 hours. After treatment, cytosolic and particulate fractions were isolated, and analyzed by Western blot for Bax and cytochrome c proteins. The protein of β‐Actin served as control for cytoplasmic fractions, cytochrome oxidase IV (Cyto ox IV) was used as the control of mitochondrial fractions. Protein intensities were calculated using ImageJ and the folds of change were as listed [Color figure can be viewed at wileyonlinelibrary.com]

The most of Bax is present in the cytosol, when apoptotic signaling is initiated; Bax undergoes a conformational rearrange and changes into a mitochondrial membrane associated protein.^28^ To further verify that the overexpression and relocation of Bax to mitochondria leads to mitochondrial depolarization and stimulation of caspases activities, we prepared subcellular fractions and determined the expression of Bad and cytochrome c in mitochondria and cytosol fractions by Western blot assay (Figure 5). We used β‐actin as control proteins for cytoplasmic fractions and cytochrome oxidase IV (Cyto ox IV) were used as the comparable controls of mitochondrial fractions. When cells were treated with 800 μM NGEN, Bax translocalized to mitochondria and induced the discharge of cytochrome c into cytosol. Therefore, our data demonstrated that NGEN induces a mitochondria‐mediated caspases dependent apoptosis in A549 lung adenocarcinoma cells.

## 4 DISCUSSION

NGEN is a flavonoid and has been shown to exhibit antioxidant effects with poor direct free radical scavenging activity; but NGEN holds the potential to activate the internal antioxidant system, which exhibitions clear hepatoprotective properties.^4^ Functionally, NGEN reduces free radicals like ROS and enhances antioxidant activity such as catalase, superoxide dismutase, glutathione in chronic diseases, including neurodegenerative disease, cardiovascular disease, diabetes, pulmonary disease, cancer and nephropathy.^3^ On the contrary, NGEN has been demonstrated to increase ROS/ER stress in a dose‐dependent manner with activation of mitochondrial apoptotic pathways and lead to death of endometriosis cells.^29^ Previously, NGEN also has been suggested to activate apoptosis in human epidermoid carcinoma A431 cells^30^ and prostate cancer cells.^11^ Therefore, NGEN possesses both chemoprevention benefits and therapeutic antitumor potential, though the individual effect may be dependent on various tissues and cells as well as NGEN concentration.

**FIGURE 6:**
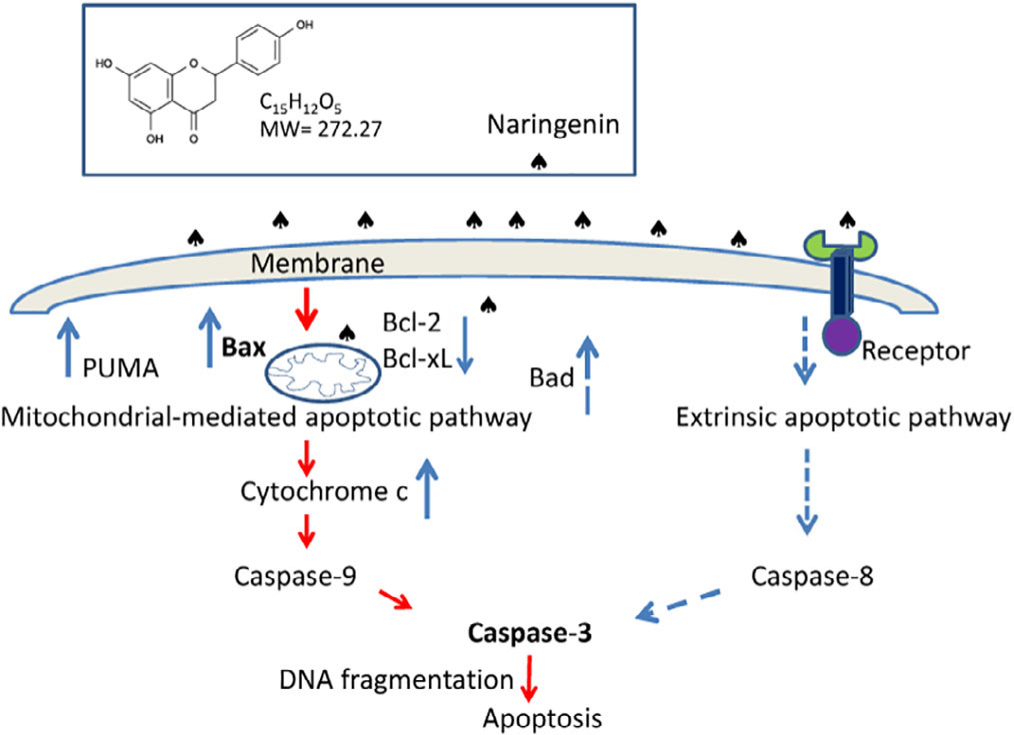
Hypothetical model of the mechanisms associated with NGEN‐induced apoptosis in lung cancer cells. The solid blue arrows indicate upregulation or downregulation of expression, the dashed blue arrows indicate possible links and the red arrows indicate the sequence of events [Color figure can be viewed at wileyonlinelibrary.com]

The molecular mechanism of NGEN‐induced cell death on lung cancer cells was investigated in the present study. According to our data (Figures 1A and 4A), low concentration of NGEN (lower than 200 μM) provide a growth inhibition effect and a high NGEN concentration (800 μM) induces apoptotic cell death. Furthermore, high concentrations of NGEN may increase ROS^30^ and activate the mitochondrial apoptotic pathway by unbalancing the Bcl‐2 group members, antiapoptotic Bcl‐2, Bcl‐xL and those proapoptotic Bax and PUMA expression, leading to Bax translocation and Bad cleavage. To conclude from our data, we have proposed a model in Figure 6 that illustrates how NGEN induces lung cancer cell death via caspases dependent pathways. The expression levels of antiapoptotic Bcl‐2 and Bcl‐xL were reduced, while those of proapoptotic Bax, and PUMA were increased. Bax translocated to mitochondria subsequent to a rapid loss of mitochondrial membrane potential. Thereafter, the movement of cytochrome c from the mitochondria to the cytosol was detected. In addition, overexpression of Bax may activate caspase‐3^31^ through the complex formation of apoptosomes (multi‐protein units containing of cytochrome c, Apaf‐1, procaspase‐9, and dATP).^16^ PUMA is transactivated by p53 to transduce death signals primarily to the mitochondria and indirectly resists all recognized antiapoptotic Bcl‐2 group members to induce mitochondrial abnormality and caspase activation.^25^ Human lung cancer A549 cells contain a wild‐type p53 that is able to induce PUMA activation upon NGEN treatment.

The minimal activation of caspase‐8 by a high concentration of NGEN was detected, along with inhibition of caspase‐8 increased cell survival with NGEN treatment (Figure 3A,B). It seems that the receptor‐mediated extrinsic apoptotic pathway could be involved in NGEN‐induced apoptosis. Indeed, a previous report has demonstrated that a low dose of NGEN (100 μM) enhances the expression of death receptor 5 and upregulates TRAIL‐induced apoptosis in A549 cells. Importantly, treatment with low dosage of 100 μM NGEN and 100 ng/mL TRAIL for 12 hours, alone or in combination, did not affect expression levels of Bcl‐2, Bcl‐xL, and Bax proteins.^20^ This observation is also supported by our data of Figure 4A. The levels of Bcl‐2, Bcl‐xL, and Bax proteins were not markedly altered when A549 cells were treated with 200 μM NGEN for 48 and 72 hours. Altogether, low concentrations of NGEN may induce death receptor 5 without mitochondria alteration; however, high concentrations of NGEN could trigger apoptosis through the intrinsic mitochondria‐mediated caspases activation signaling pathway.

## 5 CONCLUSION

In summary, the present study contributes original results to demonstrate the mechanism of NGEN‐mediated apoptosis in human lung cancer cells and shows that cell death is due to mitochondrial membrane dysfunction, nuclear condensation and DNA fragmentation. We also demonstrated that cytotoxic NGEN induced the lung cancer cell cycle sub‐G1 phase and caspase‐3 activity. Our data rule out possible antioxidant activity of NGEN that may interfere with cancer management and further support the potential of NGEN can be a compound with chemotherapeutic and cytostatic activity in human lung cancer treatment. Therefore, NGEN may be useful for further practice in drug improvement.

## CONFLICT OF INTEREST

The authors declare that there is no conflict of interest.

## AUTHOR CONTRIBUTIONS

Win‐Long Lu analyzed the results and writing the manuscript. Chang‐Tze Ricky Yu performed experimental assay and analyzed the results. Hsiu‐Man Lien analyzed the results and performed manuscript revision. Shur‐Hueih Cherng and Gwo‐Tarng Sheu designed and performed experimental assay and control data quality. All authors have read and confirmed the final manuscript.

## Data Availability

The materials included in this manuscript, may be made freely available to any researchers who wish to use them for non‐commercial purposes, while preserving any necessary confidentiality and anonymity.
